# Heart Rate Variability Biofeedback Based on Slow-Paced Breathing With Immersive Virtual Reality Nature Scenery

**DOI:** 10.3389/fpsyg.2019.02172

**Published:** 2019-09-20

**Authors:** Johannes Blum, Christoph Rockstroh, Anja S. Göritz

**Affiliations:** Department of Occupational and Consumer Psychology, Institute of Psychology, Albert-Ludwigs-Universität Freiburg, Freiburg, Germany

**Keywords:** virtual reality, nature environment, heart rate variability, biofeedback, relaxation self-efficacy, mind wandering, attentional focus, attentional resources

## Abstract

This study investigated the benefits of using a virtual nature environment to administer immersive heart rate variability biofeedback (HRV-BF) based on slow-paced breathing. We compared the virtual reality (VR)-based HRV-BF with a standard implementation in a randomized controlled experiment with 60 healthy employees. After a cognitive stress induction, the participants performed a single-session of HRV-BF before repeating the cognitive stressor task. VR-based versus standard HRV-BF was comparable in terms of biofeedback performance (cardiac coherence and cardiac vagal tone). However, the VR-based implementation buffered perceived stress in the subsequent stressor task, increased relaxation self-efficacy more, reduced mind wandering, helped participants focus on the present moment, and helped preserve attentional resources. Potential long-term effects and generalizability need to be assessed in future research.

## Introduction

### Heart Rate Variability Biofeedback

In a healthy heart, the intervals between adjacent heartbeats (i.e., interbeat intervals) show certain fluctuations, called *heart rate variability* (HRV). Functionally, HRV allows the organism to adapt to changing exogenous and endogenous demands (for an overview, see Acharya et al., 2006; Shaffer et al., 2014). Physiologically, measures of HRV represent parasympathetic nervous system activity within cardiac regulation [Task Force of The European Society of Cardiology and The North American Society of Pacing and Electrophysiology (Task Force), 1996]. As the vagus nerve is the main contributor to the parasympathetic nervous system, certain measures of HRV (e.g., root mean square of successive differences; RMSSD) can be seen as an index for cardiac vagal tone (Laborde et al., 2017). Notably within the context of stress management, cardiac vagal tone can be considered responsible for cognitive, affective, social, and health-related self-regulatory mechanisms [Task Force of The European Society of Cardiology and The North American Society of Pacing and Electrophysiology (Task Force), 1996; Berntson et al., 1997; Porges, 2007; Thayer et al., 2009; Laborde et al., 2017, 2018]. In line with that, low HRV is associated with an increased overall mortality risk (Thayer and Lane, 2007; Thayer et al., 2010) and various psychological disorders (Kemp et al., 2010; Kemp and Quintana, 2013; Chalmers et al., 2014) and has been suggested as a *transdiagnostic biomarker of psychopathology* (Beauchaine and Thayer, 2015). By contrast, high levels of HRV are associated with cardiovascular health and resilience (Thayer et al., 2012; Walker et al., 2017).

A range of HRV-related theories surround cardiac vagal tone and its psychophysiological implications (for a comprehensive review, see Shaffer et al., 2014), focusing on different benefits, such as improved executive cognitive performance and emotional health regulation (neurovisceral integration model; Thayer et al., 2009), better social functioning (polyvagal theory; Porges, 2007) or improved regulation of energy exchange (biological behavioral model; Grossman and Taylor, 2007). The most recent among those theories is the *Vagal Tank Theory* (Laborde et al., 2018). Integrating previous theoretical considerations from a neurophysiological perspective (Thayer et al., 2009) and a cognitive psychological perspective (Baumeister et al., 2018), it posits that cardiac vagal tone can be depleted and replenished and provides an integrative psychophysiological index of self-regulation. Consequently, the theory separates resting levels of cardiac vagal tone from reactivity and recovery levels. While depleting factors may decrease cardiac vagal tone momentarily, replenishing factors may boost it at the reactivity level and might help build higher long-term baseline levels (recovery level), which in turn represent improved psychophysiological self-regulation (Laborde et al., 2018).

A potential way to replenish cardiac vagal tone and help develop higher long-term HRV is biofeedback. In biofeedback, an individual’s physiological state is measured via different pathways or parameters (e.g., heart rate, electrodermal activity, brain activity; for an overview of different biofeedback approaches, see Yu et al., 2018) and fed back in real time. The immediate feedback helps the individual gain voluntary control over the respective physiological process and induces favorable changes. Heart rate variability biofeedback (HRV-BF) specifically aims at increasing individual cardiac vagal tone. Trainees receive feedback regarding their current HRV and, depending on the exact implementation, learn to apply different techniques in an attempt to increase their individual HRV. The effectiveness of HRV-BF in treating stress-related disorders and symptoms has been established in reviews and meta-analyses (Wheat and Larkin, 2010; Gevirtz, 2013; Goessl et al., 2017; Kennedy and Parker, 2018; Yu et al., 2018).

A common HRV-BF implementation utilizes the physiological link between the breath and the heart. Through regulatory physiological mechanisms in the autonomic nervous system, inhaling increases and exhaling decreases the heart rate. This effect, called *respiratory sinus arrhythmia* (RSA), is one of the main components of HRV (Hayano et al., 1996) and is most present with low respiratory frequencies (Song and Lehrer, 2003). Slow-paced breathing provides an accessible way of increasing HRV as well as improving psychological well-being and emotion regulation (Zaccaro et al., 2018). In such implementations of HRV-BF based on respiration regulation (also referred to as RSA biofeedback), trainees learn to utilize slow breathing maneuvers to improve HRV (Lehrer and Gevirtz, 2014). Corresponding parameters are retrieved from interbeat intervals and fed back to the trainee in real time.

A prominent feedback parameter in recent studies of respiration-based HRV-BF is *cardiac coherence* (for a recent review, see Yu et al., 2018). It refers to the degree of resonance between respiration and heart rate oscillations. High coherence implies a close coupling of heart rate and breathing and is marked by sine-like oscillations around 0.1 Hz in the time series of interbeat intervals. From a physiological point of view, cardiac coherence is beneficial because it makes the autonomic nervous system work most efficiently (see also the biological behavioral model, Grossman and Taylor, 2007). Heart rate, gas exchange, and baroreflex are coupled; the available oxygen is thus optimally leveraged (Lehrer and Gevirtz, 2014). According to the resonance model (Lehrer and Gevirtz, 2014), cardiovascular resonance (i.e., high cardiac coherence) requires slow-paced breathing at around six breath cycles per minute (i.e., 0.1 Hz), which represents the body’s resonance frequency (Vaschillo et al., 2006). Coherence can be assessed in real time via spectral analysis of a short moving series of interbeat intervals by estimating the relative power in the band around the resonance frequency (c.f., McCraty et al., 2009). The present study utilizes cardiac coherence as a meaningful and flexible feedback parameter that can be integrated with different biofeedback settings or implementations.

### Immersive Nature Environments in Heart Rate Variability Biofeedback

Common implementations of HRV-BF make use of abstract graphical or numerical indicators and charts to present the feedback parameters in a comprehensive way (Peek, 2017; Yu et al., 2018). In the following, we use the term *standard HRV-BF* to refer to these well-established implementations. Although standard HRV-BF appears to be effective, there are a range of common hindrances that might prevent deliberate practice and thus limiting desirable training effects. Since HRV-BF has some complexity, the feedback parameters might not be meaningful to the user or worse, might be hard to gain control over, which can lead to negative feedback experiences and negative affect. Consequently, users might be frustrated and lack motivation to continue the training. Moreover, HRV-BF requires a sustained focus on the breath and the feedback, a self-regulatory process that taps into attentional resources. In case of failed self-regulation, the user may experience distracting thoughts and inability to continuously pace their breathing. In case of successful self-regulation, attentional resources might be diminished after the training (c.f., Baumeister et al., 2018). While training the self-regulatory capacity is part of HRV-BF and will improve over time, there is still the risk of demotivation or negative learning experiences.

We believe that these hindrances can be overcome, at least in part, by embedding HRV-BF in a setting that provides a comforting and enjoyable environment, promotes sustained attention, and offers immersive feedback elements. Research on the use of simulated environments for stress reduction and relaxation has demonstrated the feasibility of virtual reality (VR; i.e., computer-generated) nature environments (de Kort et al., 2006; Anderson et al., 2017; Liszio et al., 2018; White et al., 2018). The positive effect of exposure to nature is subject to the *Attention Restoration Theory* (Kaplan, 1995). This theory posits that the voluntary attentional resources of an observer can be restored by involuntary attention toward an environment that provides a feeling of *being away* from daily routines, that is *expansive* and *coherent*, that elicits *soft fascination*, and that is *compatible* with the observer’s preferences and goals (Kaplan, 1995). Therefore, virtual nature environments appear to be a suitable setting for HRV-BF as they replenish attentional resources in a comforting and relaxing way and provide plenty of opportunities for immersive and meaningful feedback elements. In the following, we use the term *VR-based HRV-BF* to refer to implementations of HRV-BF that make use of computer-generated virtual environments, independent of the presentation medium (e.g., computer screen, spatial arrangement of several screens, head-mounted display). We argue that virtual nature environments have the potential to enhance HRV-BF in the following ways.

#### Relaxation

In HRV-BF, trainees typically receive both positive and negative feedback. Negative feedback might be perceived as a stressor that triggers distracting associations and impedes proper breathing (c.f., Boiten et al., 1994). To buffer adverse effects, it is mandatory to establish a comforting biofeedback setting. Using virtual nature environments in HRV-BF makes it possible to shape and control a biofeedback environment that promotes relaxation. Empirically, nature environments are among the most relaxing environments. Even short exposure to nature has been shown to reduce stress and restore productivity (Kaplan, 1995; Bowler et al., 2010; Berto, 2014; Ohly et al., 2016). This also applies to virtual substitutes of nature (Villani and Riva, 2012; Annerstedt et al., 2013; Gromala et al., 2015; Serrano et al., 2016; Andersen et al., 2017; Anderson et al., 2017). Consequently, in a VR-based HRV-BF, the training can take place in a virtual nature environment that leverages the restorative potential of nature. This may foster a relaxed attitude in the trainee and buffer negative effects of potential stressors.

*Hypothesis 1* (H_1_): A VR-based HRV-BF implementation promotes relaxation more than a standard HRV-BF implementation.

#### Relaxation Self-Efficacy

HRV-BF in general may increase trainees’ relaxation self-efficacy because success in producing the target behavior is immediately fed back and thus perceivable (e.g., Giardino et al., 2004; Paul and Garg, 2012; Teufel et al., 2013). In a VR-based HRV-BF, changes in the target biological parameters can be mapped to changes in the virtual nature environment. It can be argued that altering all of one’s surroundings in VR provides a more powerful and meaningful experience than altering a numerical value or a line on a chart as in standard biofeedback. Thus, VR-based HRV-BF should foster participants’ confidence in a stronger way. It should be noted that stronger feedback could potentially backfire if a trainee fails to produce the desired outcomes. However, the feedback does not need to be implemented as powerful or drastic in its negative form (i.e., negative feedback may only consist of reverting the positive feedback), and the comforting environment should buffer negative effects as described above. Furthermore, with VR-based HRV-BF, the trainee may more likely learn on a metaphorical level that a change of their own emotional and physical state can alter the way they perceive the environment. Also, for that property, in a VR-based HRV-BF, a comparable level of success in the biofeedback should have a stronger impact on the trainee’s confidence in their ability to reduce stress and alter biological functions than in a standard HRV-BF.

*Hypothesis 2* (H_2_): A VR-based HRV-BF implementation fosters relaxation self-efficacy more than a standard implementation.

#### Mind Wandering and Focus on the Present Moment

In a VR-based HRV-BF, the user is confronted with immersive virtual stimuli capable of inducing a *sense of presence*, that is the feeling of actually being inside the virtual environment (Sanchez-Vives and Slater, 2005; Cummings and Bailenson, 2016). Thus, virtual nature environments may reduce mind wandering in the form of distracting thoughts because the trainee’s attention is involuntarily drawn toward the engaging virtual environment. Consequently, by means of providing a salient environment, VR promotes an experiential focus on the present moment.

This mechanism has been utilized to help overcome acute, procedural, and chronic pain (Won et al., 2017) and to help practice mindfulness meditation (Gromala et al., 2015; Kosunen et al., 2016; Navarro-Haro et al., 2017). Applied to HRV-BF, virtual nature environments provide task-related and salient feedback, thereby reducing distractions in terms of both intrusive thoughts unrelated to the task and negative thoughts concerning one’s own biofeedback performance. As a result, participants in a VR-based biofeedback training should be less distracted and find it easier to focus on the present moment and the desired behavior.

*Hypothesis 3a* (H_3a_): A VR-based HRV-BF implementation leads to a greater focus on the present moment than a standard implementation.

*Hypothesis 3b* (H_3b_): A VR-based HRV-BF implementation leads to less mind wandering than a standard implementation.

#### Attentional Resources

According to the neurovisceral integration model (Thayer et al., 2009; Smith et al., 2017), cardiac vagal tone is positively associated with the performance of executive functions. In support of that, cognitive performance in a modified Stroop task has been found to be increased after a short-term HRV-BF (Prinsloo et al., 2011). This positive effect is attenuated by the requirement to continuously regulate the attentional focus toward the feedback stimuli and the desired physiological changes, which depletes attentional resources. A VR-based HRV-BF should alleviate this issue for two reasons. First, as argued above, the trainees’ need to actively inhibit distracting thoughts is reduced, thereby facilitating directed attention to the biofeedback and thus conserving this valuable resource (for a conception of directed attention as a finite resource, see Baumeister et al., 2018). Second, research on the Attention Restoration Theory (Kaplan, 1995; Kaplan and Berman, 2010) has shown that nature exposition helps regain concentration and acts as an antidote to attentional fatigue (for a review, see Ohly et al., 2016). Therefore, HRV-BF in a virtual nature environment should be less demanding in terms of consumption of attentional resources and more restorative in terms of concentration.

*Hypothesis 4* (H_4_): A VR-based HRV-BF leaves the user with a higher level of attentional resources than a standard implementation.

#### Heart Rate Variability

Lastly, following the reasoning above, the benefits of a VR-based implementation might be observable in terms of actual biofeedback performance. A more focused and relaxed paced-breathing exercise might have a stronger effect on RSA and fosters higher cardiac coherence. Moreover, and in line with the vagal tank theory (Laborde et al., 2018), virtual nature embedded biofeedback could boost cardiac vagal tone (as indexed by RMSSD) at the reactivity level due to the replenishing nature of the virtual environment and the stronger focus on the task itself with less distracting thoughts.

*Hypothesis 5* (H_5a_): A VR-based HRV-BF yields higher cardiac coherence than a standard implementation.

*Hypothesis 5* (H_5b_): A VR-based HRV-BF yields higher cardiac vagal tone than a standard implementation.

## Materials and Methods

### Participants and Design

Participants were recruited in local companies via professional as well as personal networks. There was no monetary reward for participation. Exclusion criteria were cardiovascular disease, psychological disorders, epilepsy, severely impaired balance, smoking, and regular use of medication as well as any other untreated medical condition. Participants were instructed not to consume caffeine or to smoke or exercise on the day of the experiment. A total of 60 healthy participants took part in the study. All of them were (self-)employed with at least 20 weekly working hours (*M* = 38.2, SD = 9.5). Thirty-one (51.7%) were women. The average age was 33.5 years (SD = 9.4). Most participants had no prior experience with biofeedback (86.7%).

The study was conducted as a double-blind, randomized, controlled, between-subjects, laboratory experiment. Participants were randomly assigned to one out of two conditions, a standard HRV-BF treatment (Standard-BF, *n* = 29) or a VR-based HRV-BF treatment (VR-BF, *n* = 31). The computerized randomization was performed for each participant individually (i.e., no block randomization), which resulted in a minor group size difference (29 vs. 31 participants). Nevertheless, the two conditions were comparable as regards age, gender distribution, work hours, and biofeedback experience (all *p* ≥ 0.448). The between-subjects design was necessary to rule out effects of training as well as a large halo effect of the novel VR implementation which would both have been present in a direct per person comparison of the two implementations in a within-subjects design.

Dependent variables were heart rate, subjective relaxation, relaxation self-efficacy, mind wandering, focus on the present moment, and attentional resources. Furthermore, we analyzed participants’ success in increasing HRV through biofeedback to check the compliance with the feedback protocol and to control for potential differences in short-term HRV between the two conditions. Additionally, we assessed the participants’ overall experience in terms of liking, reported compliance, and discomfort. To control for potential confounders, we assessed self-reported meditation habit, respiration exercise experience, and physical fitness. There were no differences between the experimental conditions on any of these variables (all *p* ≥ 0.268).

### Biofeedback Treatment

Both conditions received a single session of HRV-BF for a duration of 10 min. The duration was chosen following previous research on short-duration HRV-BF (e.g., Prinsloo et al., 2011; Kim et al., 2013). Participants were asked to practice slow diaphragmatic breathing at a frequency of six breaths per minute (i.e., 0.1 Hz) guided by an auditory pacemaker. The auditory pacemaker consisted of a recording of a male person performing slow and relaxed breathing (six cycles per minute) with a natural variation that allowed for the participants to anticipate the start and end of each breathing phase^1^. Ten complete breathing cycles were recorded and played back repeatedly. The audio file was identical in both conditions. An inhalation to exhalation ratio of 5:5 with no pause between the breathing phases was chosen in accordance with prior research findings indicating that HRV is most increased when inhalation and exhalation phases are equal in length (Lin et al., 2014).

Continuous measurement and real time spectral analyses of interbeat intervals were applied to obtain a feedback parameter for assessing heart coherence. The interbeat intervals were captured wirelessly via the validated and reliable *Polar H7* chest strap (Gillinov et al., 2017; Plews et al., 2017), which was directly connected to a custom man-in-the-middle application on a Windows 10 computer via Bluetooth Low Energy. For every incoming measurement, artifacts were corrected in case of a deviation greater than 350 ms from the local moving average of the last 10 data points and replaced by the respective mean. All valid intervals within the last 60 s were cubic-spline interpolated and resampled at 2 Hz (evenly spaced samples within the data series, c.f., Singh et al., 2004); the DC component was removed from the data series (detrending the data series to exclude trend heart rate); a *Hann-Window* was applied (tapering the amplitudes of the end points of the data series to prevent leakage and improve the time resolution, c.f., Singh et al., 2004); and a forward *Fast-Fourier* transformation was performed, which yields estimates of *power spectral densities* of different frequencies within the input signal. To compute the feedback parameter, we performed the following four steps (adapted from McCraty et al., 2009): (1) compute the spectral power between 0.04 and 0.26 Hz (total power), (2) identify the highest peak in the band between 0.04 and 0.26 Hz, (3) compute the spectral power in a window 0.015 Hz above and below the highest peak (peak power), and (4) calculate the feedback parameter: coherence = peak power/total power.

Besides the continuous coherence feedback parameter that ranged from 0 to 1, we computed a dichotomous feedback parameter by applying a threshold of 0.5 to the coherence value. A value of 0.5 and above signifies that at least half of the analyzed spectral power falls into a small frequency range, resulting in a sine-like HRV waveform. This indicates a close coupling of heart rate and breathing oscillations in that the heart rate rhythmically increases on inhalation and decreases on exhalation.

The two conditions differed in the implementation of the feedback. In the Standard-BF condition, participants received abstract feedback in the form of graphical geometrical indicators. The continuous feedback parameter was represented by the blue fill-amount of a horizontal bar in the center of the screen. The dichotomous feedback was visualized by a circular color indicator below the bar that turned from red to green whenever the threshold was surpassed. Through a pair of over-ear headphones, ambient instrumental background music as well as the auditory pacemaker was played back, with a volume ratio that put a clear emphasis on the pacemaker and allowed participants to easily discern the pacemaker from the subtle musical background.

**Figure 1:**
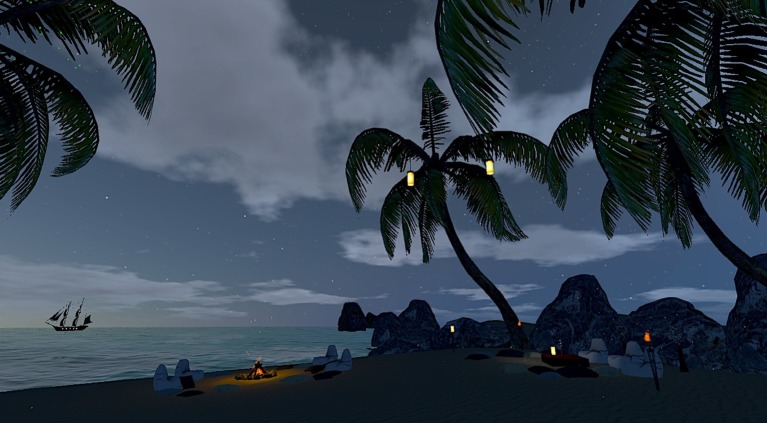
Screenshot of the virtual environment in the VR-BF condition.

In the VR-BF condition, participants experienced a virtual beach scenery at sunset (Figure 1). We rigorously designed the scene to be in conjunction with the characteristics described in the Attention Restoration Theory (Kaplan, 1995). The scene comprised a private beach setting to create a feeling of being away. It included palms, rocks, several light sources, and a campfire to offer various stimuli that gently draw the attention and allow for soft fascination. The scene was set with a view of an ocean to let the eyes wander into the expansive distance. The visuals were accompanied by a corresponding soundscape with ocean and campfire sounds as well as the same ambient instrumental background music as in the Standard-BF. To integrate the restorative environment and the biofeedback task seamlessly, we embedded the feedback into the scenery by utilizing environmental features as the feedback stimuli. The continuous feedback parameter was bound to the cloud coverage in the evening sky; the better the parameter, the clearer the star-spangled sky. The dichotomous feedback was visualized by several lamps as well as a campfire that were lit whenever the threshold was surpassed and vice versa.

The design of the virtual environment was meant to provide an experience as immersive and realistic as possible. To optimize the experience, we used a contemporary head-mounted display (HMD, *Oculus Rift CV1*) to present the environment. As the Standard-BF condition was delivered via a regular computer screen (*Dell U2415*), the study cannot entirely rule out possible effects of the presentation format. Nevertheless, we chose to use an HMD in the VR-BF condition because the restorative effects of nature can only be plausibly expected in a simulation if the user experiences a high sense of presence, thus experiencing the environment instead of merely viewing it. This can be best guaranteed by using an HMD. On the contrary, it is not feasible to use an HMD to deliver two-dimensional graphical feedback. The pixel density and visual clarity of HMD is not well-suited for the sustained and comfortable perception of text and simple graphical stimuli. On a more practicable note, the presentation of simple stimuli via an HMD that adds weight to the participants’ head and may strain the eyes due to the proximity of the display, seems to add an unnecessary downside to a standard biofeedback implementation. The fact that the VR-based treatment actually included these downsides was an inevitable necessity of the more immersive delivery format and needed to be accounted for in the experimental comparison of the two implementations. Furthermore, we reduced the potential advantage of the HMD in physically blocking external stimuli as much as possible. We used the same over-ear headphones to block auditory distractors in both conditions. In the standard implementation condition, we shielded the participants from visual distractors via spatial separator walls and the use of neutral colors and stimuli surrounding the computer screen.

### Instruments

The state version of the *State Trait Anxiety Inventory* (STAI-S; Spielberger et al., 1983) was the subjective measure of participants’ relaxation. The questionnaire contains 20 statements (sample item: “I feel calm”) with four-point rating scales (1 = *not at all* to 4 = *very much so*).

To measure relaxation self-efficacy, we used 10 self-phrased items combined to a scale (see the Supplementary Material), due to lack of a validated scale. We followed Bandura (2006) recommendations for phrasing the items (sample item: “How confident are you right now that you can control your worries and fears, even when you are stressed out?”) as well as for the building of the entire scale. With each item, participants were asked to indicate their degree of approval on a visual analogue scale (VAS; 0 = *not confident at all* to 1 = *completely confident*; physical size: 1,000 px, ~27 cm).

To measure mind wandering during the treatment, we used the *Cognitive Interference Questionnaire* (CIQ; Sarason et al., 1986). The CIQ consists of the 10-item task-related interference subscale (sample item: “I thought about how poorly I was doing.”) and the 11-item task-irrelevant interference subscale (sample item: “I thought about personal worries.”). Participants rated their experience during the treatment on five-point rating scales (1 = *never* to 5 = *very often*).

Focus on the present moment was assessed via the *State Mindfulness Scale* (SMS; Tanay and Bernstein, 2013), which comprises the 15-item mindfulness of mind subscale (sample item: “I felt closely connected to the present moment”) and the six-item mindfulness of body subscale (sample item: “I clearly physically felt what was going on in my body”). With each item, participants indicated their degree of approval (1 = *not at all* to 5 = *very much*) with regard to their experience during the treatment.

To objectively assess attentional resources, we used a computerized modified Stroop task (c.f., MacLeod, 1991). Participants were instructed to react to the font color of a word-stimulus as quickly and accurately as possible by pressing a corresponding key with the index and middle finger of their left and right hand. With congruent stimuli, the font color of the word matched its semantic meaning, with incongruent stimuli, font color, and semantic meaning diverged. Congruent and incongruent stimuli were presented randomly within one single block. Furthermore, we instructed participants to count all occurrences of the word “gray.” Gray font color was not used. Thus, “gray” was always an incongruent stimulus. These control items were meant to ensure active processing of the meaning of each word. To sum up, the stimuli consisted of the words “blue,” “yellow,” “green,” “red,” and “gray” with the possible font colors blue (rgb: 11, 141, 255; luminance: 60), yellow (rgb: 255, 191, 0; luminance: 78), green (rgb: 0, 199, 60; luminance: 62), or red (rgb: 255, 61, 36; luminance: 57). Stimuli were presented in uppercase letters and a physical size of 1.5 × 4 cm on a dark gray background in the center of a screen placed roughly 70 cm away from the participants. Participants first performed a 40-trial exercise block with colored rectangles in order to memorize the color-key mapping. The subsequent test block consisted of 160 trials (72 congruent, 72 incongruent, and 16 control items). Stimuli were presented randomly; the color-word combinations were balanced. Within each trial, a fixation cross (400 ms) was followed by a blank screen (400 ms) and the actual stimulus (2 s response time window). An error message appeared in case of delayed or wrong participant reaction. Both the reaction times and the error rate were gathered. In the end, the participants were asked to indicate the total number of control items.

A Polar H7 chest strap captured the heart rate (beats per minute) as well as interbeat intervals (milliseconds). Cardiac coherence was calculated following McCraty et al. (2009) as described above. The Kubios HRV software (Tarvainen et al., 2014) was used to calculate RMSSD, which is an established indicator of cardiac vagal tone [Task Force of The European Society of Cardiology and The North American Society of Pacing and Electrophysiology (Task Force), 1996; Berntson et al., 1997; Laborde et al., 2017].

Furthermore, to assess the participants’ overall experience of the biofeedback treatment, we asked them to rate their degree of liking (“How much did you enjoy the experience?”), effort to comply with the biofeedback task (“How hard did you try to breathe deeply and rhythmically?”), and perceived discomfort (“Did you experience any kind of discomfort?”) on VAS (0 = *not at all*, 1 = *very much*; physical size: 1,000 px, ~27 cm).

### Procedure

**Figure 2:**
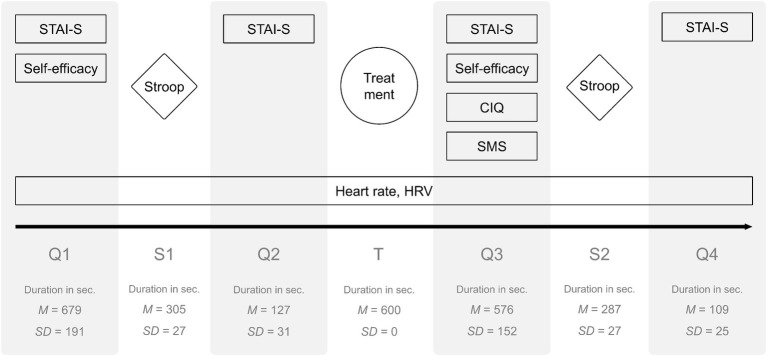
Phases of the experiment. Q, questionnaire; S, Stroop; T, treatment.

At the outset, all participants gave written informed consent and put on the chest strap, which then measured heart data continuously. Next, participants were guided through the different phases of the experiment (Figure 2) by a dedicated computer application. STAI-S (subjective relaxation) was measured four times (Q1: baseline; Q2: after the first Stroop task; Q3: after the treatment; Q4: after the second Stroop task), relaxation self-efficacy two times (Q1: baseline; Q3: after the treatment), and CIQ (mind wandering) and SMS (focus on the present moment) once (Q3: after the treatment). Heart data were captured continuously and later aggregated for each phase. The Stroop task was administered two times, before the treatment (S1) and after the treatment (S2). The task served as both a measure of attentional resources and a stressor. A female condition-blind experimenter assisted the participants with adjusting the chest strap and ensured correct acquisition of physiological data.

### Analysis of Heart Data

The continuous heart data series was separated into the seven experimental phases; the parameters were computed for each phase. Therefore, the aggregations are based on varying numbers of data points (for the mean duration of each phase, see Figure 2). However, the treatment phase, which is most crucial in terms of the hypothesis, was of equal length (10 min) for all participants.

As HRV data are often non-normally distributed and may require log-transformation (Laborde et al., 2017), we checked for normal distribution within all experimental cells. In some instances, the data were skewed. To rule out methodological artifacts, we performed all HRV-related analyses with and without prior natural-log-transformation. The patterns were highly comparable in all cases and neither *p* nor effect sizes differed notably in any case. To apply the least possible and necessary amount of mathematical manipulation to the original data, we decided to go with the untransformed dataset.

## Results

### Relaxation (H_1_)

**Figure 3:**
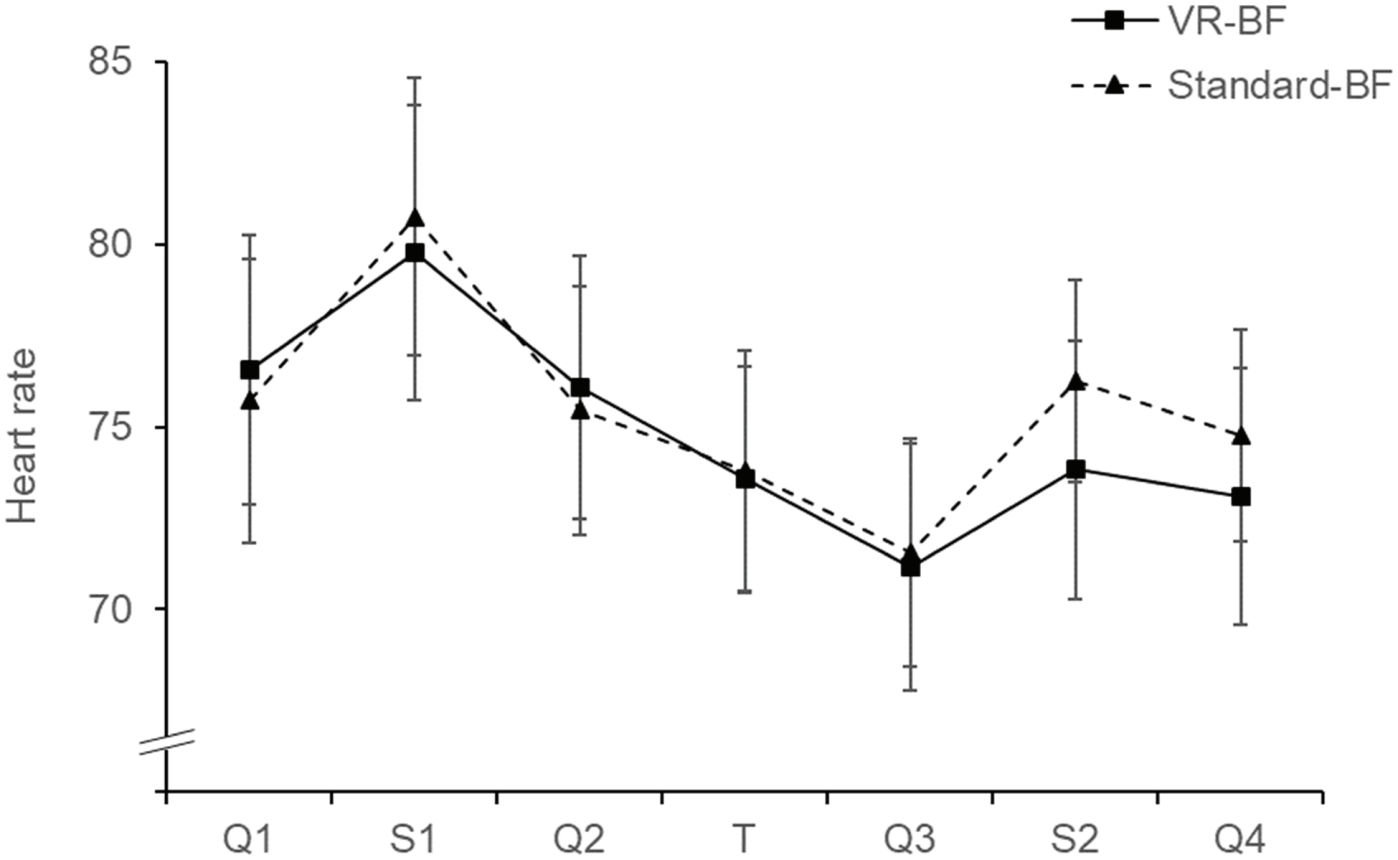
Mean heart rate (bpm) by condition. Error bars represent 95% confidence intervals (CI).

**Figure 4:**
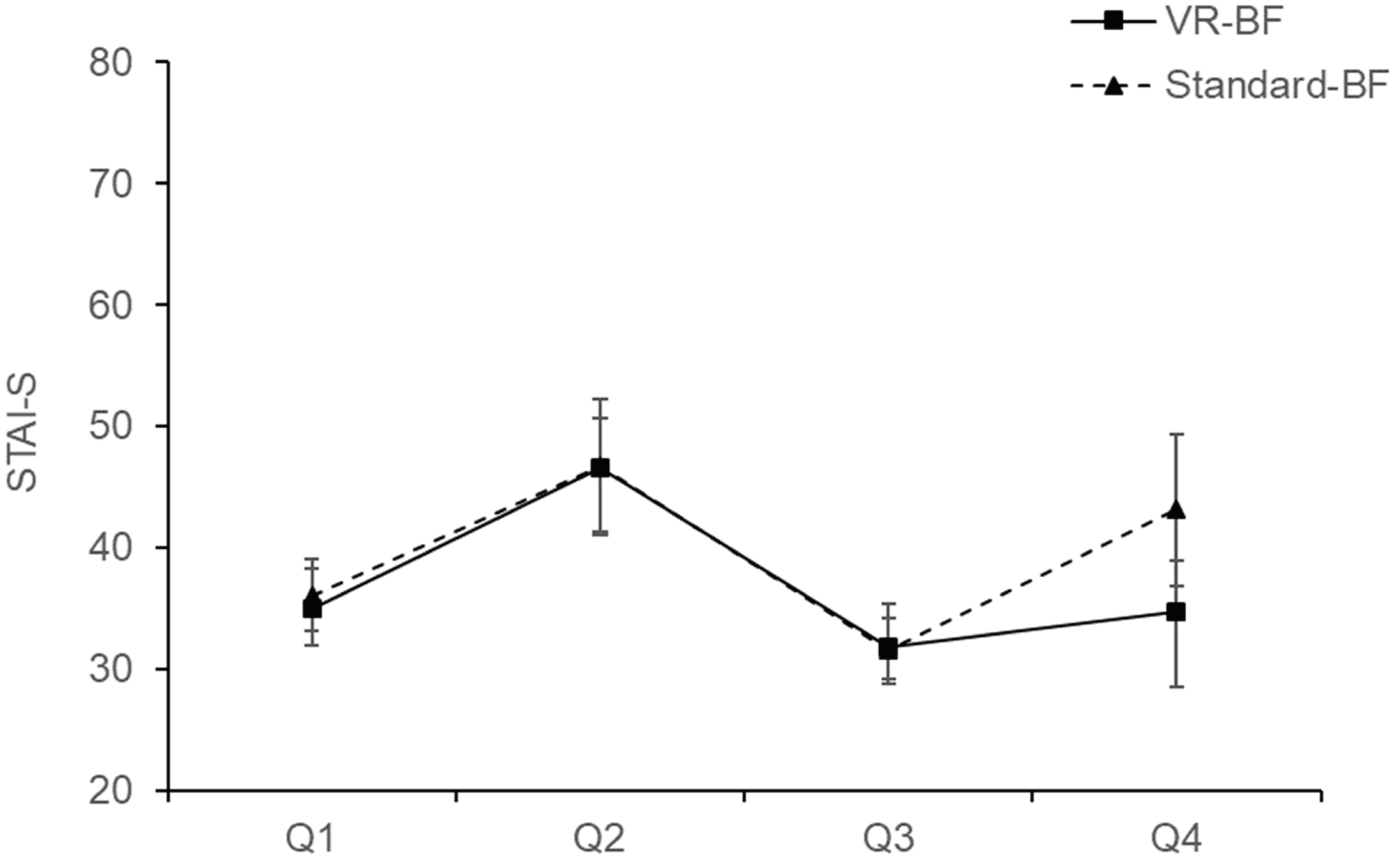
Mean STAI-S (sum score) by condition. Error bars represent 95% CI.

To investigate treatment-specific effects on relaxation (H_1_), we computed two separate analyses on heart rate (Figure 3) and STAI-S (Figure 4). The mixed ANOVA on heart rate with the factors Time (levels: Q1, S1, Q2, T, Q3, S2, Q4; within-subjects) and Condition (VR-BF vs. Standard-BF; between-subjects) showed no Condition × Time interaction, *F*(3.233, 187.506) = 1.439, *p* = 0.230, and no effect of Condition, *F*(1, 58) = 0.074, *p* = 0.787. The analysis revealed an effect of Time, *F*(3.233, 187.506) = 30.870, *p* < 0.001, $\eta_{p}^{2}$ = 0.347. Bonferroni-adjusted pairwise comparisons revealed higher heart rate during both Stroop tasks (S1 and S2) compared to the respective preceding phase (both Δ_mean_ ≥ 3.676, both *p* < 0.001), and a stepwise reduction in heart rate from S1 to Q2 to T to Q3 (all Δ_mean_ ≥ 2.086, all *p* ≤ 0.002).

Cronbach’s *α* of the STAI-S were *α* = 0.88 in Q1, *α* = 0.95 in Q2, *α* = 0.92 in Q3, and *α* = 0.97 in Q4. The mixed ANOVA on STAI-S with the factors Time (levels: Q1, Q2, Q3, Q4; within-subjects) and Condition (VR-BF vs. Standard-BF; between-subjects) revealed a Condition × Time interaction, *F*(1.991, 115.463) = 3.192, *p* = 0.045, $\eta_{p}^{2}$ = 0.052. Simple main effect analyses revealed no differences between VR-BF and Standard-BF at Q1, Q2, and Q3 (all *p* ≥ 0.602), whereas at Q4 VR-BF scored lower (*M* = 34.77, SD = 11.39) than Standard-BF (*M* = 43.10, SD = 16.42), *F*(1, 58) = 5.269, *p* = 0.025, $\eta_{p}^{2}$ = 0.083, indicating that VR-BF participants did not deteriorate in relaxation due to the second Stroop task as much as Standard-BF participants. Furthermore, there was an effect of Time, *F*(1.991, 115.463) = 31.645, *p* < 0.001, $\eta_{p}^{2}$ = 0.353. Bonferroni-adjusted pairwise comparisons showed higher values after each Stroop (Q2 and Q4) compared to before (both Δ_mean_ ≥ 3.825, both *p* ≤ 0.005) and a treatment induced reduction from Q2 to Q3 (Δ_mean_ = 14.959, SE = 1.863, *p* < 0.001).

To sum up, both Stroop tasks reduced and both treatments increased relaxation. There was no between-groups difference in relaxation during the treatment. However, the relaxation in the VR-BF was not lowered as much by the second Stroop task as in the Standard-BF. Therefore, H_1_ is partly confirmed.

### Relaxation Self-Efficacy (H_2_)

**Figure 5:**
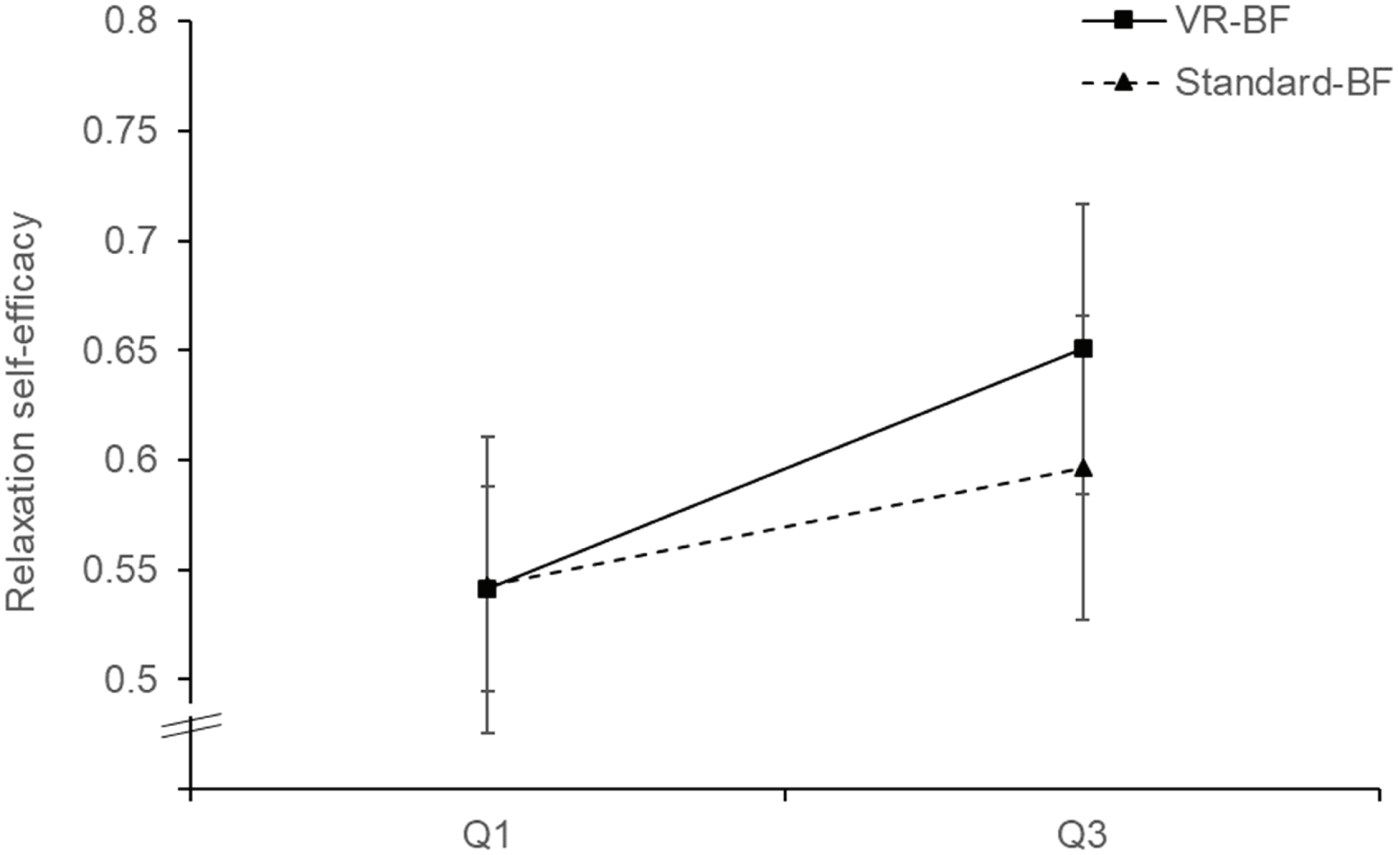
Relaxation self-efficacy by condition. Error bars represent 95% CI.

Cronbach’s *α* of the relaxation self-efficacy scale were *α* = 0.89 in Q1 and *α* = 0.94 in Q3. To investigate whether VR-BF improved relaxation self-efficacy more than Standard-BF (H_2_), we conducted a mixed ANOVA with the factors Time (Q1 vs. Q3; within-subjects) and Condition (VR-BF vs. Standard-BF; between-subjects). There was a Condition × Time interaction, *F*(1, 58) = 4.547, *p* = 0.037, $\eta_{p}^{2}$ = 0.073. Relaxation self-efficacy increased more in VR-BF (Δ_mean_ = 0.110) as compared to Standard-BF (Δ_mean_ = 0.054), thus supporting H_2_ (Figure 5).

### Focus on the Present Moment (H_3a_)

**Figure 6:**
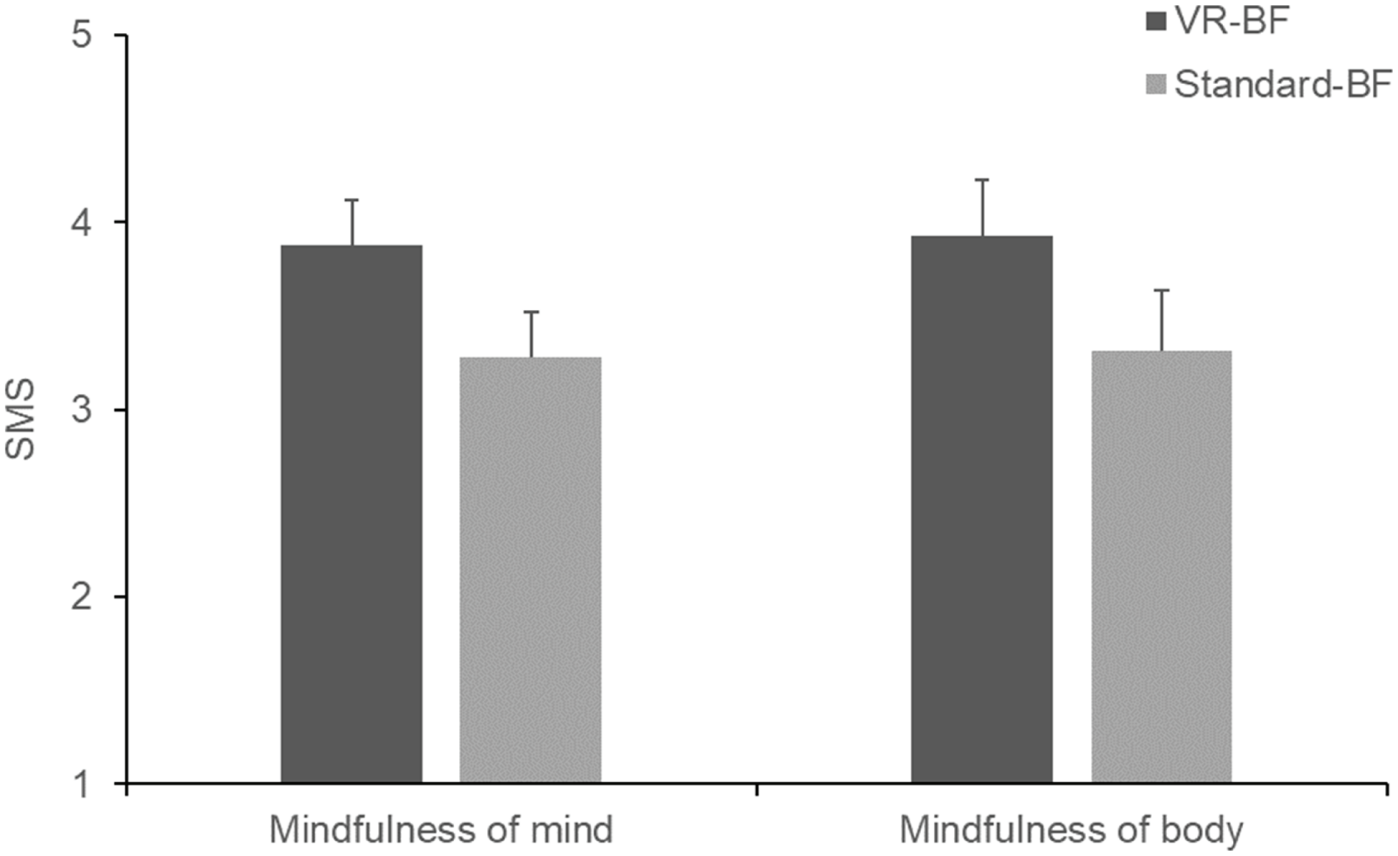
Mean state mindfulness of mind and body by condition. Error bars represent 95% CI.

Cronbach’s *α* of the SMS were *α* = 0.92 with the state mindfulness of mind subscale and *α* = 0.82 with the state mindfulness of body subscale. To test whether VR-BF resulted in a stronger focus on the present moment than Standard-BF (H_3a_), we computed a separate one-way ANOVA for each SMS subscale (Figure 6). Participants scored higher on the state mindfulness of mind subscale in VR-BF (*M* = 3.88, SD = 0.64) compared to Standard-BF (*M* = 3.28, SD = 0.80), *F*(1, 58) = 10.234, *p* = 0.002, $\eta_{p}^{2}$ = 0.150. Likewise, participants scored higher on the state mindfulness of body subscale in VR-BF (*M* = 3.92, SD = 0.65) compared to Standard-BF (*M* = 3.32, SD = 0.83), *F*(1, 58) = 10.040, *p* = 0.002, $\eta_{p}^{2}$ = 0.148. Therefore, H_3a_ is confirmed.

### Mind Wandering (H_3b_)

**Figure 7:**
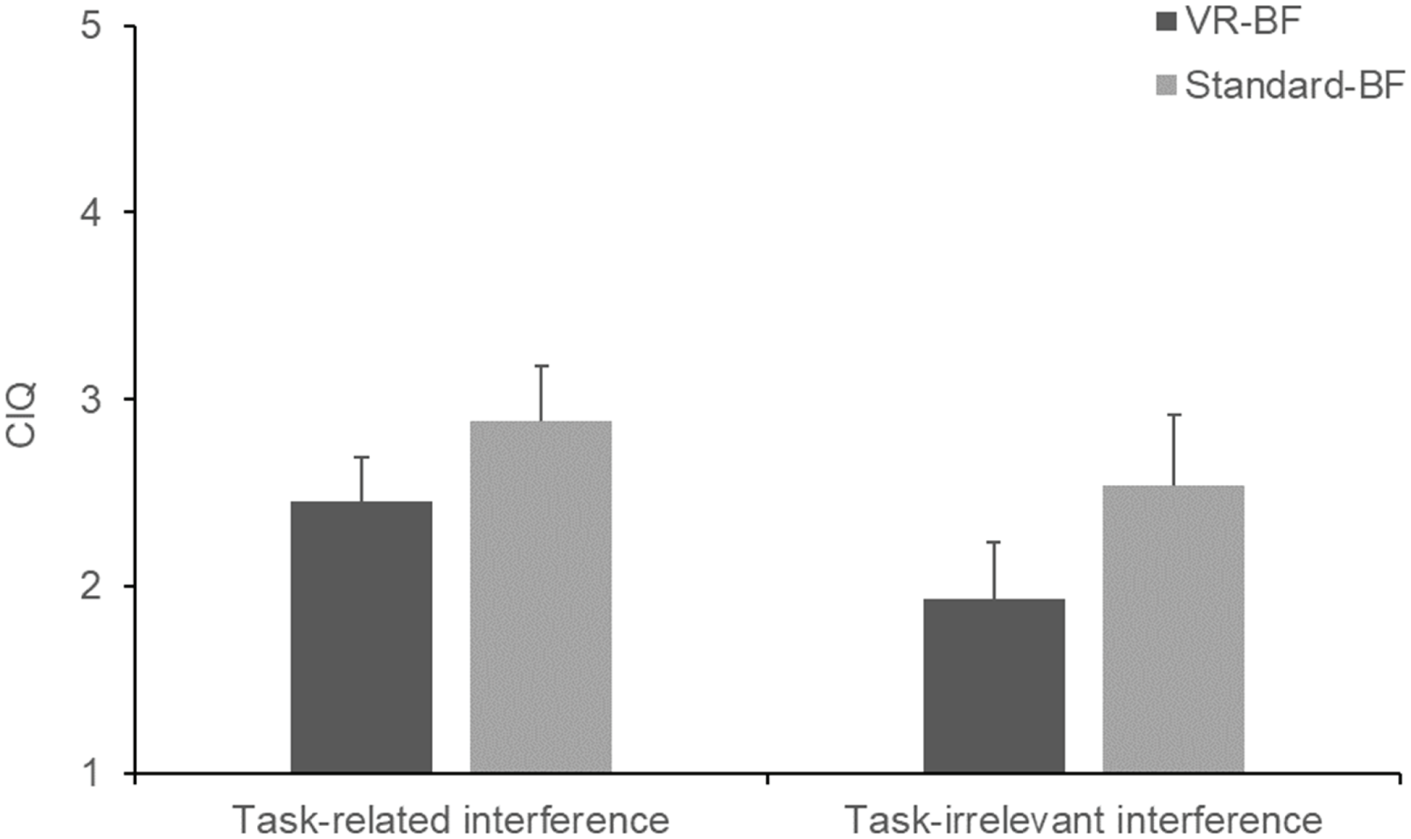
Mean task-related and task-irrelevant interference by condition. Error bars represent 95% CI.

Cronbach’s *α* of the CIQ were *α* = 0.81 with the task-related subscale and *α* = 0.91 with the task-irrelevant subscale. To test whether VR-BF gave rise to less mind wandering than Standard-BF (H_3b_), we computed separate one-way ANOVAs for each CIQ subscale (Figure 7). Participants scored lower on the task-related subscale in VR-BF (*M* = 2.45, SD = 0.66) compared to Standard-BF (*M* = 2.89, SD = 0.79), *F*(1, 58) = 5.404, *p* = 0.024, $\eta_{p}^{2}$ = 0.085. Likewise, participants scored lower on the task-irrelevant subscale in VR-BF (*M* = 1.93, SD = 0.80) compared to Standard-BF (*M* = 2.54, SD = 1.01), *F*(1, 58) = 6.580, *p* = 0.013, $\eta_{p}^{2}$ = 0.102. Therefore, H_3b_ is confirmed.

### Attentional Resources (H_4_)

**Figure 8:**
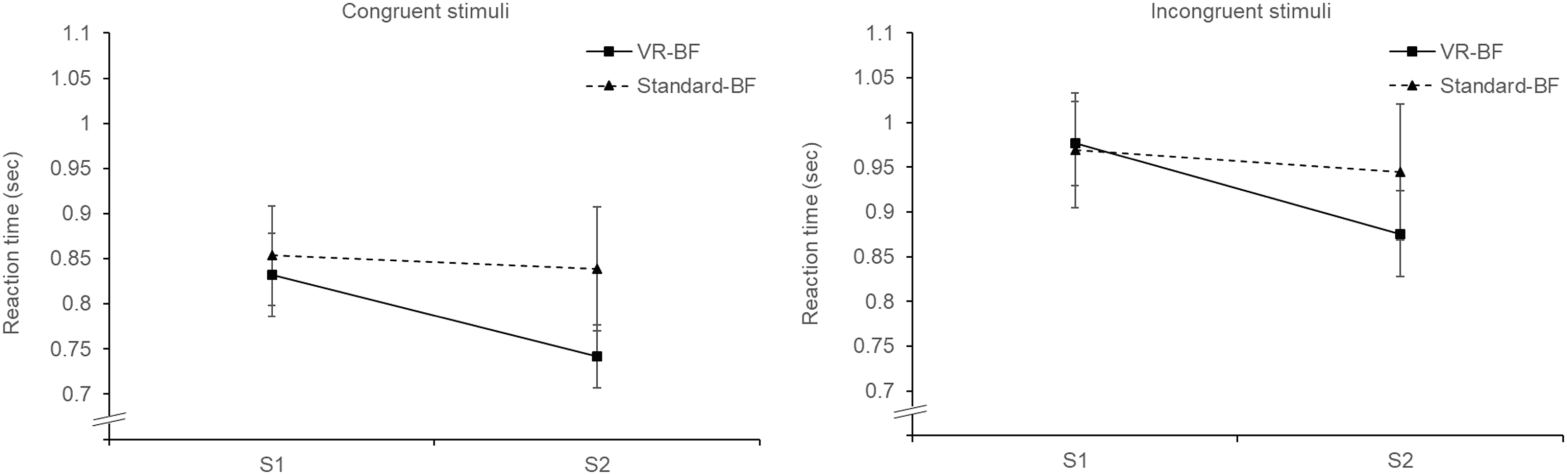
Mean reaction times (in seconds) for congruent and incongruent stimuli by condition. Error bars represent 95% CI.

Overall, error rates were low (4.61% for S1, 2.6% for S2). Likewise, participants in both groups showed little absolute deviance in indicating the correct number (= 16) of control items (S1: *M* = 1.85, SD = 1.90; S2: *M* = 1.00, SD = 1.47). This shows that the participants took the Stroop task seriously and focused on accuracy rather than speed. Therefore, to investigate the effect of the treatment on attentional resources (H_4_), we analyzed reaction times (Figure 8). For each participant, we aggregated the reaction times of the correctly answered trials (Ratcliff, 1993), excluding the few control items to ensure equal numbers of congruent and incongruent trials. Since intra-participant reaction time distributions are typically skewed, we used the median as an aggregator instead of the arithmetic mean because it is more robust against outliers and asymmetrical distributions (Rousselet and Wilcox, 2018), which has also been empirically demonstrated with the Stroop task (Singh and Göritz, 2019).

We conducted a mixed ANOVA for reaction time with the three factors Time (S1 vs. S2; within-subjects), Trial type (congruent trials vs. incongruent trials; within-subjects), and Condition (VR-BF vs. Standard-BF; between-subjects). As expected, the analysis showed an effect of Time, *F*(1, 58) = 19.371, *p* < 0.001, $\eta_{p}^{2}$ = 0.250, and an effect of Trial type, *F*(1, 58) = 295.944, *p* < 0.001, $\eta_{p}^{2}$ = 0.836, but no effect of Condition, *F*(1, 58) = 1.619, *p* = 0.208. This indicates that there was a learning effect from the first (*M* = 0.905, SD = 0.019) to the second Stroop task (*M* = 0.843, SD = 0.020), that there was an overall Stroop effect (congruent trials: *M* = 0.816, SD = 0.133; incongruent trials: *M* = 0.941, SD = 0.147), and that the groups did not differ *per se*. There was no Trial type × Condition interaction, *F*(1, 58) = 3.847, *p* = 0.055, i.e., the Stroop effect was comparable in the two groups. However, there was a Time × Condition effect, *F*(1, 58) = 8.397, *p* = 0.005, $\eta_{p}^{2}$ = 0.126, revealing that the reduction in reaction times over time was greater with VR-BF (Δ_mean_ = 0.095) than with Standard-BF (Δ_mean_ = 0.028). This effect did not depend on the type of trial as there was no Time × Trial type × Condition interaction, *F*(1, 58) = 0.005, *p* = 0.944. In conclusion, participants in the VR-BF performed better after the treatment than participants in the Standard-BF, which supports H_4_.

### Heart Rate Variability (H_5_)

**Figure 9:**
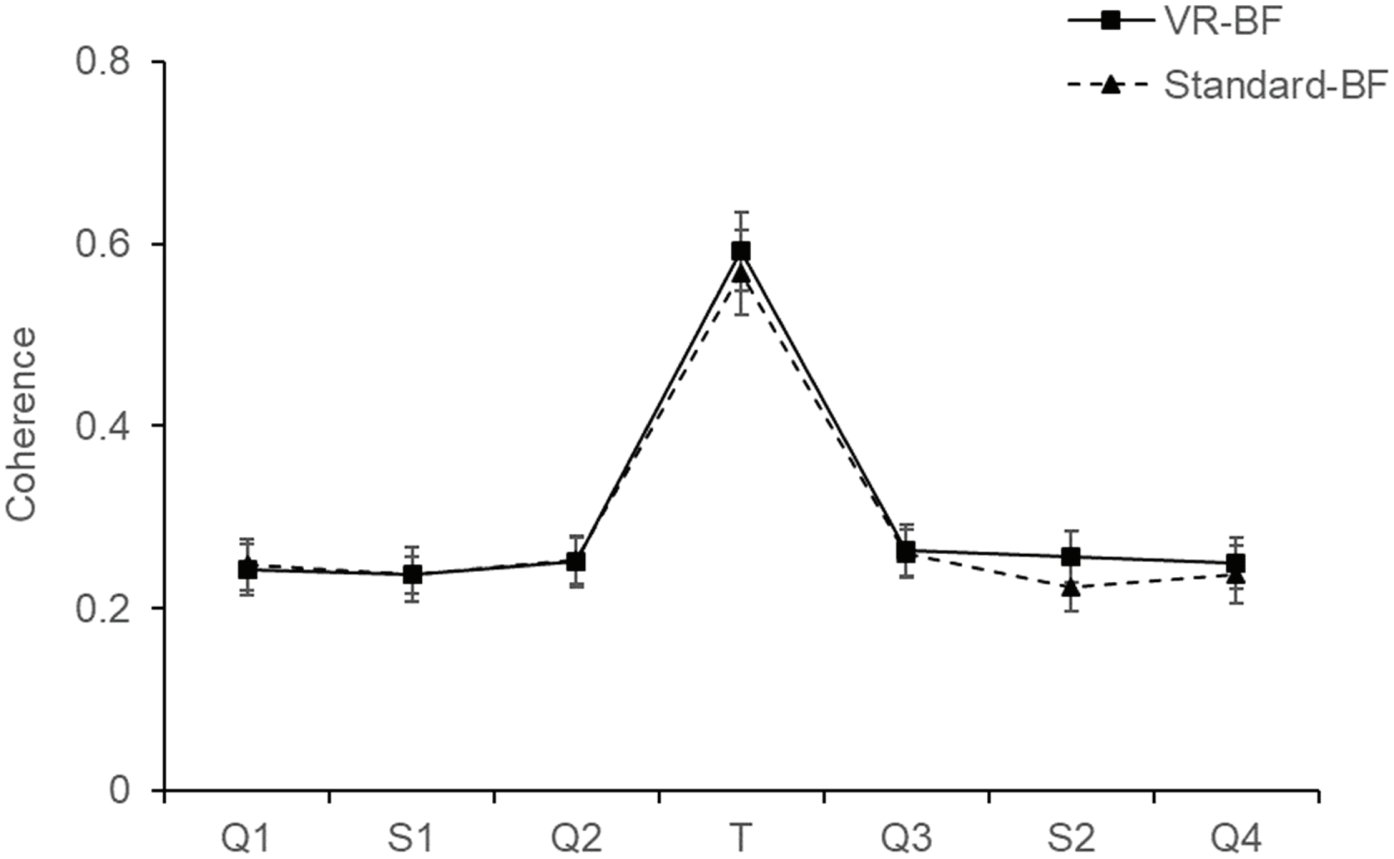
Cardiac coherence by condition. Error bars represent 95% CI.

**Figure 10:**
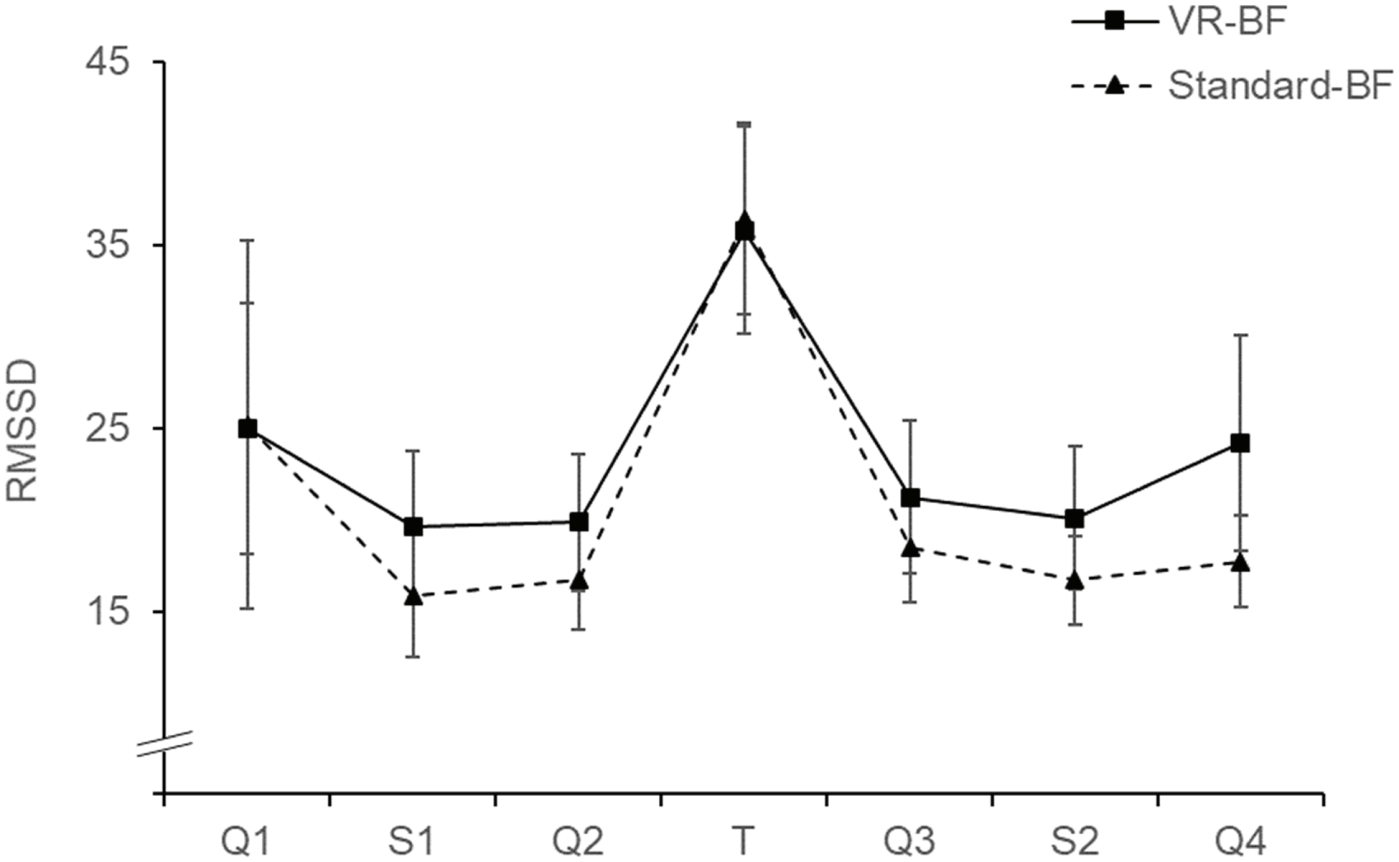
RMSSD (ms) by condition. Error bars represent 95% CI.

To investigate the effect of the treatments on participants’ cardiac coherence (H_5a,_ Figure 9) and cardiac vagal tone (H_5b,_ Figure 10), we conducted separate mixed ANOVAs for cardiac coherence and RMSSD with the factors Time (levels: Q1, S1, Q2, T, Q3, S2, Q4; within-subjects) and Condition (VR-BF vs. Standard-BF; between-subjects).

The ANOVA on cardiac coherence revealed no Condition × Time interaction, *F*(3.407, 197.597) = 0.715, *p* = 0.561, and no effect of Condition, *F*(1, 58) = 0.470, *p* = 0.496. There was an effect of Time, *F*(3.407, 197.597) = 237.972, *p* < 0.001, $\eta_{p}^{2}$ = 0.804. Bonferroni-adjusted pairwise comparisons revealed significantly higher cardiac coherence during the treatment compared to any other phase (all Δ_mean_ ≥ 0.318, all *p* < 0.001), with no differences among the other phases (all *p* ≥ 0.155).

For RMSSD, there was no Condition × Time interaction, *F*(2.442, 141.662) = 0.709, *p* = 0.520, and no effect of Condition, *F*(1, 58) = 1.471, *p* = 0.230. The analysis revealed an effect of Time, *F*(2.442, 141.662) = 20.865, *p* < 0.001, $\eta_{p}^{2}$ = 0.265. Bonferroni-adjusted pairwise comparisons revealed increased RMSSD during the treatment compared to any other phase (all Δ_mean_ ≥ 11.055, all *p* ≤ 0.020), with no differences among the other phases (all *p* ≥ 0.263).

In conclusion, participants in both the VR-BF as well as the Standard-BF increased cardiac coherence and cardiac vagal tone substantially during the biofeedback task. There were, however, no significant differences between the experimental conditions. Therefore, H_5a_ and H_5b_ are rejected.

### Overall Experience

The reported effort to comply with the biofeedback task as well as perceived discomfort did not differ between the groups (both *p* ≥ 0.202). However, VR-BF (*M* = 0.741, SD = 0.255) showed higher liking ratings than Standard-BF (*M* = 0.569, SD = 0.257), *F*(1, 58) = 6.716, *p* = 0.012, $\eta_{p}^{2}$ = 0.104.

## Discussion

The present study tested whether a VR-based HRV-BF implementation outperforms a standard implementation on physiological, affective, and cognitive outcome measures. We hypothesized that the VR-based implementation improves relaxation (H_1_) and relaxation self-efficacy (H_2_), increases a focus on the present moment (H_3a_), decreases mind wandering (H_3b_), helps conserve attentional resources (H_4_), and improves cardiac coherence (H_5a_) as well as cardiac vagal tone (H_5b_). Overall, results support the assumption that VR enhances HRV-BF.

A beneficial effect of the VR-based implementation as regards relaxation (H_1_) was confirmed only partly. While both implementations were effective in increasing relaxation in terms of both physiological and subjective measures, there was no treatment-specific difference. Hence, VR did not increase the immediate relaxing effect of HRV-BF, which contradicts H_1_. While our study showcases the relaxing effect of HRV-BF, it is possible that our implementation of virtual nature does indeed not elicit greater relaxation *per se*. Alternatively, the fact that VR can have an arousing effect due to the high degree of immersion (c.f., Riva et al., 2007; Felnhofer et al., 2015) could have neutralized a relaxing effect of the virtual nature. Such a suppressing effect would be expected to steadily decline with repeated exposure to VR. Additionally, the measures used, heart rate and STAI-S, might not have entirely captured the calming effects of virtual nature. Heart rate primarily measures arousal, and the STAI-S focusses on anxiety. Instruments that explicitly assess different aspects of affect and mood (e.g., Positive and Negative Affect Scale, Crawford and Henry, 2004; Multidimensional Mood Questionnaire, Steyer et al., 1997) might have revealed more. However, in the VR implementation, subjective relaxation was less reduced by the second Stroop task compared to the standard implementation. This points toward a buffer effect of VR-based HRV-BF, which protects against subsequent stressors, thus supporting H_1_. Given that both groups adequately relaxed during the treatment, this stress-shielding effect seems more beneficial than a momentary effect, as it points toward an effect that exceeds the duration of the treatment. The stress-shielding applied particularly to the psychological domain. While heart rate increased slightly less in the VR-based implementation, this effect did not reach a conventional level of statistical significance.

As expected, the VR-based implementation led to a greater increase in relaxation self-efficacy (H_2_), more focus on the present moment (H_3a_; state mindfulness of body and mind assessed via SMS), and less mind wandering (H_3b_; task-related and task-irrelevant cognitive interference assessed via CIQ), supporting all three hypotheses. As regards relaxation self-efficacy, the greater increase in the VR-based implementation cannot be attributed to a higher level of biofeedback success, as both groups performed comparably. This greater increase also indicates that the stronger feedback in the form of environmental changes did not backfire in the case of negative feedback. However, as participants generally were able to achieve and maintain a high level of HRV during the treatment and therefore mostly received positive feedback, this cannot be concluded with certainty. Likewise, the rich virtual environment did not impair participants’ concentration on the task, indicating that the design of the virtual environment succeeded in avoiding a seductive details effect (c.f., Rey, 2012). On the contrary, the immersive experience safeguarded from internal and external distractions. Furthermore, these properties of VR-based HRV-BF are beneficial as they help maintain sustained concentration as well as motivation and may thus influence biofeedback success (e.g., Khazan, 2015; Weerdmeester et al., 2017). In the long run, this could boost the efficacy of HRV-BF as compared to standard implementations.

In line with H_4_, participants in the VR-based implementation showed a better performance in the Stroop task than participants in the standard implementation, in that their reaction times improved significantly more from the first Stroop task to the second. The greater speed did not come at the cost of accuracy, as error rates were comparable. Interestingly, the greater improvement in reaction times applied to both congruent and incongruent stimuli. There was no treatment-specific effect on the reduction of the Stroop effect. This could have been caused by the combination of both stimuli types within a single block, increasing the processing demand for congruent stimuli and implementing a task-switching exercise (Singh and Göritz, 2019). However, the presence of an overall Stroop effect indicates that there was still a clear difference in processing difficulty between congruent and incongruent stimuli. Hence, it is more likely that the VR-based implementation affected a more general directed attention, necessary for both types of stimuli, instead of a more specific inhibitory control, which is required in the inhibition of the prepotent response in incongruent items. The effect is in line with Attention Restoration Theory (Kaplan, 1995; Ohly et al., 2016), which posits the restorative effect of nature experiences on directed attention.

Lastly, the discussed benefits of the VR-based implementation did not manifest in a superior biofeedback performance (cardiac coherence H_5a_; cardiac vagal tone, H_5b_). The lack of an interaction effect indicates that there was no implementation-specific difference in the change of the respective parameters, neither during the biofeedback task nor subsequently. Notably, the VR-based implementation also did not come at the cost of reduced biofeedback success. Both groups performed adequate slow breathing and substantially increased cardiac coherence and cardiac vagal tone (RMSSD) during the treatment in a comparable manner. Within the concept of vagal tank theory (Laborde et al., 2018), both feedback implementations represented comparable replenishing factors in terms of cardiac vagal tone during this reactivity phase. There was no carry-over effect to any of the subsequent experimental phases, as the investigated parameters were comparable before and after the respective treatment. As regards experimental control, the lack of a difference between the two groups in terms of cardiac coherence and cardiac vagal tone indicates that the effects regarding the other four hypotheses were not mediated by greater biofeedback success.

### Limitations and Future Research

While our findings are based on a randomized and controlled computer-based laboratory experiment with a sample consisting of employees from all walks of life, some methodological limitations could not be ruled out. Participants liked the VR biofeedback significantly better. While this is a desirable outcome, we cannot rule out that some of the effects on the subjective measures might have been tinted by a halo effect. Furthermore, we assessed relaxation self-efficacy using a custom scale of 10 self-framed items. Although we followed the best-practice recommendations by Bandura (2006) and the scale had high internal consistency (*α* ≥ 0.89), for lack of a validated scale, we could not use one. Next, our findings regarding attentional resources rely on a custom modification of the Stroop color-word task. Generalizability to other implementations of the same task as well as to different measures of attentional resources (e.g., digit span task) remains an open research question. Additionally, the paced-breathing task in this study implemented an inhalation to exhalation ratio of 1:1. Future research needs to investigate the influence of different ratios in the context of our study, given that evidence suggests a longer exhalation than inhalation when targeting increases in cardiac vagal tone (Lehrer et al., 2000; Strauss-Blasche et al., 2000; Vaschillo et al., 2006; Allen and Friedman, 2012; Lehrer and Gevirtz, 2014). Moreover, we cannot make any claims in terms of longitudinal effects. Although it seems possible that the effects would become even more pronounced over time, the single session of HRV-BF used in our study needs to be extended to a multi-session study to examine longer term effects. Furthermore, as explained in the “Methods” section, a potential effect of the technical delivery modality (HMD vs. computer screen) cannot be accounted for. Next, although assessing HRV via a chest strap has been found to be reliable in most conditions (Gillinov et al., 2017; Plews et al., 2017), it does not allow for precise artifact correction via visual inspection of the ECG signal to understand whether artifacts are due to technical or physiological issues (Berntson and Stowell, 1998; Laborde et al., 2017). In addition, the experiment did not include a dedicated HRV baseline but captured the unmanipulated HRV data in the beginning of the experiment during the first questionnaire phase. Although this appears to be a viable baseline surrogate, future research should include a proper baseline measurement. As mentioned above, the lack of anxiety assessed by the STAI-S does not directly indicate relaxation. Lastly, it is unlikely that in this early attempt on VR-based HRV-BF, we found the optimal realization. Further studies should fine tune the realization to augment its effectiveness.

## Conclusion

In our study, we investigated the benefits of implementing HRV-BF in a virtual nature scenario. Our findings suggest that a VR-based implementation increased relaxation self-efficacy, reduced mind wandering, increased focus on the present moment, and conserved attentional resources in a greater way than a standard implementation. Moreover, there was evidence suggesting that HRV-BF in VR buffers the negative impact of a subsequent stressor. These results are important and useful in terms of training motivation and practice, even though there were no differential effects of the implementations on cardiac coherence and cardiac vagal tone. Studies with multiple training sessions are needed to investigate possible long-term effects. The study offers insights as to the feasibility of using virtual nature environments in HRV-BF and paves the way for future research in this field.

## Data Availability Statement

The datasets generated for this study are available on request to the corresponding author.

## Ethics Statement

Ethical review and approval was not required for the study on human participants in accordance with the local legislation and institutional requirements. The patients/participants provided their written informed consent to participate in this study.

## Author Contributions

CR, JB, and AG developed the study concept and design. CR and JB designed the virtual environment, programmed the experiment, collected the data, and performed the data analyses. CR and JB drafted an initial version of the manuscript. AG provided revisions. All authors approved the final version of the manuscript for submission. CR and JB contributed equally to this work and share first authorship.

### Conflict of Interest

The authors declare that the research was conducted in the absence of any commercial or financial relationships that could be construed as a potential conflict of interest.

## Footnotes

^1^The corresponding audio file cannot be provided because it is proprietary and protected by copyright.
