# Supplementary material for: Heart Rate Variability Biofeedback Based on Slow-Paced Breathing With Immersive Virtual Reality Nature Scenery
Source: Front Psychol. 2019 Sep 20;10:2172. doi: 10.3389/fpsyg.2019.02172 (PMC6763967; doi:10.3389/fpsyg.2019.02172)
Supplement: Supplementary file 1 [file Table_1.docx]

Supplementary Material

# Relaxation Self-Efficacy Scale

(self-phrased following Bandura, 2006)

How confident are you right now that you can…

(Visual Analogue Scale; 0 = *not confident at all* to 1 = *completely confident*)

1. control your worries and fears, even when you are stressed out?

2. relax even when under time pressure?

3. maintain your inner balance even in times of high workload?

4. keep a cool head even under high performance pressure?

5. keep your mind calm when facing negative thoughts?

6. control your physical tension even when you are stressed out?

7. calm your body down even when under time pressure?

8. regulate your body even under high performance pressure?

9. regenerate your body even in times of high workload?

10. stay physically calm even when facing negative thoughts?
